# Time-Series Analysis of Microtopographic Evolution and Morphological Changes in Regressive Tidal Creeks via UAV-LiDAR

**DOI:** 10.3390/s26134257

**Published:** 2026-07-04

**Authors:** Juneseok Kim, Hyeyeon Yoon, Ilyoung Hong

**Affiliations:** 1 Department of Drone & GIS Engineering, University of Namseoul, Cheonan City 31020, Republic of Korea; junesoek.kim@nsu.ac.kr; 2 Ecological and Natural Map Team, National Institute of Ecology, Seocheon-gun 33657, Republic of Korea; gis82@nie.re.kr

**Keywords:** UAV, LiDAR, DEM, microtopography, tidal creek, time-series analysis, regressive tidal

## Abstract

This study conducted a six-month time-series micro-topographic analysis using high-resolution UAV LiDAR technology to precisely characterize the complex terrain changes in regressive tidal creeks within coastal wetlands. To overcome the unique challenges posed by vegetation-dense regressive tidal flats, the LiDAR Penta Return (5-pulse) mode was applied, yielding high-density point cloud data with an average of 174 pts/m^2^. The analysis successfully reproduced the bare earth surface beneath the vegetation canopy at sub-centimeter-level precision, overcoming the limitations of conventional optical surveying, and enabled quantitative detection of micro-topographic changes of ±25 cm or greater. Time-series analysis based on the DEM of Difference (DoD) revealed spatiotemporally asymmetric erosion and deposition patterns concentrated at the lower elevation zone (0.0–2.0 m) and slope boundaries of the regressive tidal creek. However, the apparent large elevation changes in the lowest, intermittently inundated creek-bed zone (including a maximum of about 3.7 m between the summer surveys, T2–T1) were found to scale monotonically with the tide level at the time of each flight, indicating that they are governed by the tide-dependent water-surface return rather than by genuine bed erosion. After excluding this water-affected zone, the consistently sub-aerial surface showed only modest net change over the six-month period, indicating that the regressive tidal creek adjusts gradually rather than through abrupt large-magnitude erosion and deposition. This study presents the essential value of high-precision time-series monitoring for assessing the geomorphic stability of coastal wetlands in an environment where extreme weather events under climate change are increasing in frequency.

## 1. Introduction

Tidal flats serve as buffer zones connecting terrestrial and marine ecosystems, representing hotspots of biodiversity with high conservation value [1,2]. These coastal depositional landforms develop extensively along coasts with large tidal ranges and are predominantly distributed along the western and southern coasts of the Korean Peninsula. In particular, the Gyeonggi Bay area hosts the most extensive tidal flat development in Korea. Sorae Wetland, located on the southeastern coast under equivalent hydrological and sedimentary conditions, lies at the boundary of Nam-dong District, Incheon Metropolitan City, and Wolgot-dong, Siheung City, Gyeonggi Province. From the Japanese colonial period until 1996, this area was home to the largest salt production facility in the country. Since its transformation into an ecological park in 2009, the site has undergone natural succession. As ground elevation has gradually increased, the area now exhibits the characteristics of a regressive tidal flat where seawater rarely inflows even during high tide, with progressive terrestrialization (landification) underway. The tidal flat’s structural configuration—where inundation of the upper flat occurs only when sea level exceeds 9 m—has produced a remarkably rare sinuous tidal creek. Quantifying the micro-topographic changes in such features is essential for elucidating the rate and pattern of terrestrialization and evaluating the impacts of recent climate change on coastal systems [3].

Topographic analyses have conventionally relied on 1:5000 scale digital topographic maps produced by the National Geographic Information Institute (NGII) or airborne Light Detection and Ranging(LiDAR) datasets [4,5]. However, these traditional approaches suffer from poor temporal immediacy and high acquisition costs. More critically, the 5 m contour interval data used in digital topographic maps simplifies fine-scale terrain records, distorting actual surface morphology. In environments such as Sorae Wetland, where vegetation is prevalent and topographic change is dynamic, it is extremely difficult to reproduce complex tidal creek structures using digital topographic maps alone. Unmanned Aerial Vehicle(UAV) imagery-based Structure-from-Motion (SfM) [6], introduced as a complementary approach, has also been reported to suffer from reduced vertical accuracy due to its inability to acquire bare earth surface information beneath the vegetation canopy [7,8].

UAV LiDAR technology has recently attracted attention as a solution to overcome these limitations [9,10]. UAV LiDAR can acquire high-density point cloud data exceeding 150 pts/m^2^, and sensors supporting multi-return functionality—such as the Zenmuse L2—can precisely penetrate through dense vegetation to reach the ground surface [11,12]. Previous studies analyzing karst terrain in Mungyeong and Danyang, North Chungcheong Province, demonstrated that UAV LiDAR achieves far superior micro-topographic reproducibility compared to digital topographic maps [11,12]. Specifically, the Root Mean Square Error (RMSE) of Inertial Measurement Unit (IMU) trajectory errors confirmed in prior research was approximately 0.004–0.006 m in both horizontal (X, Y) and vertical (Z) directions, with a final vertical accuracy of approximately 8 mm, confirming the theoretical sub-centimeter accuracy of the system [13].

Regressive tidal creeks are distinct from ordinary tidal creek systems because their hydrological connectivity with the open sea is progressively reduced as tidal flats undergo elevation increase and terrestrialization. Whereas typical tidal creeks are regularly shaped by tidal inundation, drainage, and sediment transport, regressive tidal creeks may remain relatively inactive under normal tidal conditions but respond abruptly when episodic forcing, such as heavy rainfall, storm-induced residual water-level anomalies, or concentrated runoff, exceeds a local geomorphic threshold. This can generate localized and nonlinear erosion–deposition patterns in low-elevation creek beds and channel banks.

However, previous studies have mainly addressed active tidal creek systems, UAV photogrammetry-based mapping, or single-epoch topographic surveys. The seasonal micro-topographic evolution of regressive tidal creeks in vegetation-covered coastal wetlands has therefore not been sufficiently quantified. This gap provides the rationale for applying repeated high-resolution UAV-LiDAR surveys to detect bare-earth micro-topographic changes in Sorae Wetland.

While previous studies by the present authors demonstrated the superior micro-topographic reproducibility of UAV LiDAR in karst terrain environments [11,12,13], those investigations were conducted under stable geological settings and did not address dynamic, hydrologically active coastal systems. Building on this research gap, the present study applies repeated UAV-LiDAR time-series monitoring to quantify seasonal micro-topographic changes in a vegetation-covered regressive tidal creek within a coastal wetland. By linking high-resolution UAV-LiDAR-derived DEMs with DoD analysis and tidal-condition data, this study aims to clarify how regressive tidal creeks respond to seasonal and episodic forcing in a coastal wetland undergoing terrestrialization. Unlike single-epoch terrain assessments, the six-month repeated survey design employed here enables the distinction between genuine subaerial micro-topographic change and tide-dependent apparent elevation change in intermittently inundated creek-bed zones. In doing so, this study bridges a critical methodological gap between high-precision UAV-LiDAR capabilities and the need for temporally resolved topographic monitoring in coastal wetland environments vulnerable to climate change.

## 2. Related Work

Tidal creeks are key geomorphic elements responsible for hydrological circulation and sediment transport within tidal flats. Precisely characterizing their morphology and subtle changes is essential for understanding tidal flat evolution and ecosystem dynamics [14,15,16]. However, tidal flats have limited accessibility and soft substrate conditions that make traditional in situ surveys extremely challenging. Airborne LiDAR (ALS) has been employed as an alternative [17,18,19], but the low point density resulting from high flight altitude has limited the ability to precisely represent the micro-topography of narrow, complex tidal creeks [16,20].

More recently, UAV-based Structure-from-Motion (SfM) photogrammetry has been introduced as an economical and timely approach for tidal flat monitoring [7,8,21,22,23,24]. Previous studies have demonstrated that UAV photogrammetry is effective for extracting the planimetric positions of tidal creeks through centimeter-level imagery [7,8,25,26]. However, photogrammetric methods are prone to matching errors in vegetation-dense sections or on textureless tidal flat surfaces, and critically suffer from significantly reduced vertical accuracy due to their inability to obtain bare earth surface information beneath the vegetation [7,20].

In this context, UAV LiDAR systems have emerged as the optimal alternative for analyzing morphological changes in complex micro-terrain such as vegetation-covered regressive tidal creeks [27,28,29]. Studies by Kim & Hong [13] and Kim & Hong [11] demonstrated that UAV LiDAR can penetrate vegetation through multi-return functionality to obtain actual ground elevation values, enabling Digital Elevation Model (DEM) generation with far superior precision compared to conventional photogrammetry or digital topographic maps. In analyses targeting karst terrain, UAV LiDAR successfully reproduced fine-scale relief features that were missing or distorted in digital topographic maps [11,12].

Furthermore, UAV LiDAR—by securing high-density point clouds exceeding 100 pts/m^2^—enables clear identification of boundaries in steep slopes and irregular micro-terrain. Research on automated tidal creek network extraction methods has also progressed steadily [30], and recent approaches have proposed integrating airborne LiDAR for broad-area terrain information with high-resolution UAV data for areas requiring detailed tidal creek analysis [20]. In this process, advanced filtering algorithms such as CSF (Cloth Simulation Filtering) have been developed to effectively remove vegetation noise and extract the actual depth and morphological information of tidal creeks [31,32,33,34]. Therefore, a high-resolution UAV LiDAR-based approach represents the most effective methodology for overcoming the limitations of conventional survey methods and elucidating the complex three-dimensional structure and time-series micro-topographic changes in regressive tidal creeks.

## 3. Materials and Methods

This study conducted high-resolution micro-topographic analysis using UAV LiDAR technology on tidal creeks located at Sorae Wetland, spanning Nam-dong District, Incheon Metropolitan City, and Wolgot-dong, Siheung City, Gyeonggi Province, Republic of Korea.

### 3.1. Study Site

**Figure 1:**
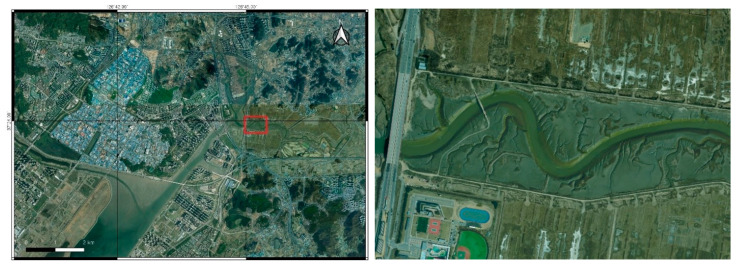
The study site (**Left**) Regional context showing Sorae Wetland at the boundary of Incheon and Siheung. (**Right**) Detailed view of the sinuous tidal channel and regressive salt marsh area.

The study site, Sorae Wetland (Figure 1), is a coastal ecosystem formed at the confluence of Jangsu Stream and Mansu Stream before their discharge into the Yellow Sea, at the boundary of Nam-dong District, Incheon, and Siheung City, Gyeonggi Province. This area was the largest salt production facility in Korea from the Japanese colonial period until 1996. Following the cessation of salt production, the site has undergone natural succession, and a unique environment of ongoing terrestrialization (landification) has continuously raised the surface elevation [35,36].

The left image of Figure 1 shows the regional context of the study area and the surface hydrological setting, while the right enlargement reveals the complex structure of the sinuous tidal channel (Sinuous Tidal Channel), the primary subject of this study. Rectilinear divisions visible throughout the image represent vestiges of former salt pans, and the area now exhibits the characteristics of a regressive tidal flat where seawater inflows only when the tide level exceeds 9 m. This morphological configuration corresponds to the regressive tidal flat model as described by Wright & Thom [37].

The distribution of deeply incised tidal creeks and surrounding halophyte vegetation visually supports this unique geomorphic structure and can generate dynamic sediment transport and micro-topographic change within the creek. However, due to these environmental constraints and the density of salt-marsh vegetation, acquiring accurate bare-earth elevation information through conventional optical surveying methods is severely limited, making precision analysis via high-resolution UAV LiDAR essential.

### 3.2. Research Methods

To understand the long-term terrestrialization trend in the study area, historical aerial photograph data from 1983 to 2017 were collected through the National Land Information Platform of the National Geographic Information Institute. The aerial photographic record was used to examine the meandering development of tidal creeks and changes in vegetation coverage, and the accelerated terrestrialization pattern observed over the past decade was utilized as background data for the UAV LiDAR precision analysis.

**Figure 2:**
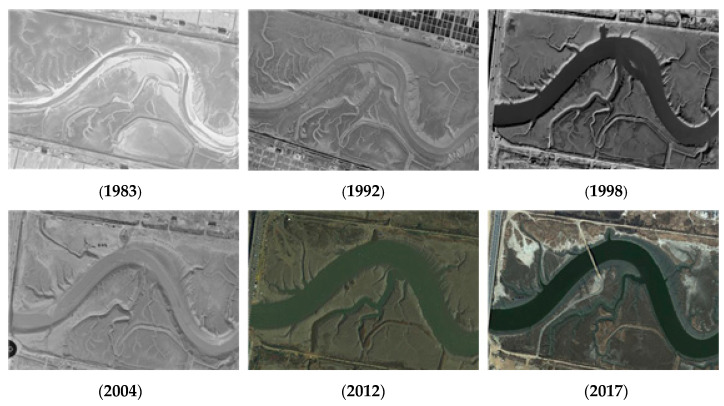
Topographical evolution and land conversion process of Sorae Wetland as documented in aerial photographs from different periods (1983~2017).

Figure 2 illustrates the geomorphic evolution of Sorae Wetland over approximately 35 years through a time series of aerial photographs spanning 1983 to 2017. The topographic change can be divided into three broad stages.

The first stage (1983–1992) was one of anthropogenic topographic maintenance. During this period, the artificial channel system and grid-pattern salt pan divisions constructed for salt production are clearly observed. The tidal creek functioned as an engineered conduit for seawater inflow and drainage, with a very limited initial distribution of vegetation on surrounding surfaces.

The second stage (1998–2004) was a transitional period of natural succession. Following the closure of the salt pans in 1996, artificial hydrological control ceased, and meandering patterns in the tidal creek began to develop dynamically. The rectilinear boundaries of the former salt pans gradually became indistinct through geomorphic adjustment, and early signs of terrestrialization were captured as halophyte communities colonized areas around the creek margins.

The third stage (2012–2017) was characterized by deepening terrestrialization and the establishment of a regressive geomorphic regime. As the tidal flat elevation continuously rose and inundation frequency declined, halophyte cover density increased dramatically. The tidal creek took on a form deeply incised relative to the surrounding terrain, forming a complex drainage network—demonstrating the typical geomorphic characteristics of a regressive tidal flat where seawater cannot freely reach the upper flat even at high tide.

**Figure 3:**
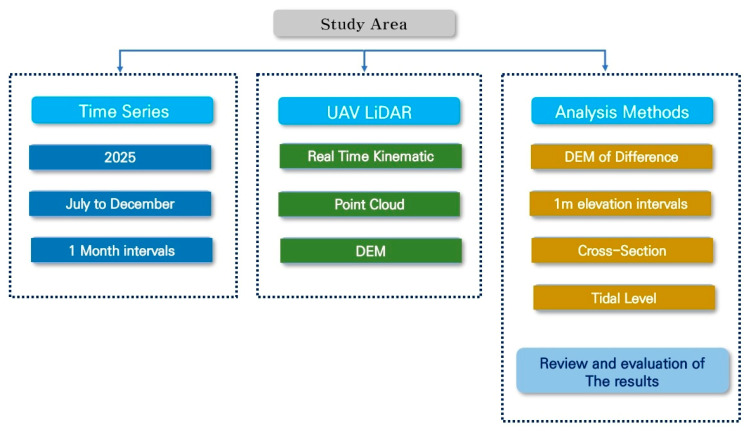
Research flow chart.

UAV LiDAR data for the study area were acquired monthly from July to December 2025 over a six-month period. Various analytical methods using high-precision LiDAR data were applied to conduct micro-topographic analysis of the regressive tidal creek. Figure 3 illustrates the research workflow.

### 3.3. UAV LiDAR Data Collection

To precisely detect subtle topographic changes in the regressive tidal creek, UAV LiDAR surveys were conducted six times monthly from July to December 2025. The survey system comprised a DJI Matrice 300 RTK platform equipped with a Zenmuse L2 LiDAR sensor. This system enables the acquisition of sub-centimeter-level precision positional data in both horizontal and vertical directions via Real-Time Kinematic (RTK) technology, enabling the collection of reliable topographic information without the need for separate ground control points (GCPs) [13,38,39,40,41].

The detailed flight plan and LiDAR settings established for precise micro-topographic measurement of the tidal creek are presented in Table 1. The flight altitude was set at 100 m, balancing micro-topographic resolution requirements with clearance from surrounding obstacles, achieving a Ground Sample Distance (GSD) of 2.93 cm. This represents the optimal altitude configuration for achieving the sub-centimeter vertical precision demonstrated in previous studies.

As Sorae Wetland is an environment densely covered by halophytic vegetation, the Penta Return (5-pulse) mode was applied to enhance ground detection rates. This mode detects the vegetation canopy top using the first return and the ground surface using the last return, demonstrating excellent performance for extracting the actual bare earth topography beneath the vegetation. The repeated scan mode was selected, which, while narrowing the scan range compared to non-repeated scanning, increases laser density for the same point and enhances data overlap, thereby maximizing topographic reproduction precision.

The dataset constructed in this study exhibits an average point density of 174 pts/m^2^—exceeding that of conventional airborne LiDAR (ALS)—and ensures sufficient data density to reproduce the steep slopes and fine steps of sinuous tidal creeks without distortion.

Table 2 summarizes the dates, temperature conditions, and meteorological status for the six UAV LiDAR observation campaigns conducted in this study. All surveys were conducted under relatively stable weather conditions with no precipitation or fog, minimizing LiDAR signal attenuation and noise generation, and ensuring the reliability of comparisons between time-series datasets.

The observation period was set from July to December 2025 to encompass the seasonal changes from summer to winter. This allowed for the simultaneous analysis of the impacts of high and low temperature periods, as well as differences in vegetation growth conditions, on the changes in microtopography of the tidal flats. Each observation was conducted while maintaining the same UAV platform and LiDAR sensor settings, and the recording of temperature and weather conditions was used to verify the consistency of the data acquisition environment, serving as supplementary material for interpreting environmental factors that may arise in the subsequent time-series DEM differencing (DoD) analysis.

## 4. Results

The UAV LiDAR-based DEM produced in this study achieved a high-resolution output with an average point cloud density of 174 pts/m^2^ and a grid spacing of 0.25 m/pixel, enabling highly precise terrain representation.

### 4.1. Data Processing and DEM Generation

To convert the raw point cloud data acquired via UAV LiDAR into a digital elevation model (DEM) capable of detailed micro-topographic analysis, a systematic data preprocessing and processing workflow was implemented. The primary objective of data processing was to construct a high-precision DEM that reliably captures subtle elevation changes (±25 cm or greater) between time-series datasets.

The raw LiDAR data acquired in the field were processed through DJI Terra (v.4.5.0) software for precise IMU trajectory correction and point cloud registration. Utilizing real-time corrected positional information from the RTK system onboard the UAV platform, sub-centimeter-level relative positional accuracy in both horizontal and vertical directions was achieved without requiring separate GCPs. Since this study specifically aims to analyze relative elevation changes (DEM of Difference, DoD) between repeatedly observed DEMs on the same datum—rather than comparing absolute elevations—the repeated surveys conducted on the same platform, with the same sensor and under identical processing conditions, provide sufficient precision for time-series comparison [13,42].

Point cloud data were classified based on attribute information including reflectivity, elevation, and return number, and ground (Ground) and non-ground (Non-ground) points were strictly separated according to the environmental characteristics of Sorae Wetland. As Sorae Wetland is an environment densely covered with halophytic vegetation where distortion of ground information is a significant concern with optical-based surveying, this study applied the Penta Return mode of the Zenmuse L2 sensor—which supports multi-return functionality—to effectively extract bare earth surface points beneath the vegetation canopy.

In the ground point extraction process, the Cloth Simulation Filtering (CSF) algorithm [31] was applied to classify ground and non-ground points and to remove vegetation and noise from the raw point cloud. CSF simulates a cloth draped over an inverted point cloud surface, iteratively adjusting the cloth to fit the terrain based on user-defined parameters. Given the dense halophytic vegetation and complex micro-topography of the tidal creek, the following parameters were configured: a cloth resolution of 0.5 m, a time step of 0.65, a classification threshold of 0.5 m, and a maximum iteration count of 500. Slope-adaptive postprocessing was enabled to account for the steep lateral banks of the sinuous tidal creek. These parameter settings were determined through iterative testing and geomorphic consistency checks based on point-cloud cross-sections, hillshade maps, and DEM surfaces, with the objective of minimizing residual vegetation noise while preserving fine-scale creek morphology. The CSF parameter settings were validated through iterative testing and visual inspection of representative geomorphic units, including creek beds, steep creek banks, and densely vegetated marsh surfaces. Classified point clouds were examined using cross-sectional profiles, hillshade maps, and DEM surfaces to confirm that vegetation artifacts were removed without over-smoothing the actual creek morphology. Residual vegetation artifacts and isolated misclassified points were manually removed where necessary, but extensive manual editing was avoided to ensure consistency across the six time-series datasets. The final parameter set was selected because it preserved narrow creek beds and steep bank features while minimizing vegetation-related noise. The high-density point cloud secured through repeated scanning accurately reproduced the steep slopes, steps, and fine channel-bed relief of the tidal creek without distortion. Through this process, a high-quality ground point cloud dataset with an average density of approximately 174 pts/m^2^ was ultimately constructed.

Based on the filtered ground point cloud, a DEM with a uniform 0.25 m × 0.25 m grid spacing was generated in DJI Terra (v.5.1.0). DJI Terra interpolates the classified ground points using a TIN-based method as its core, supplemented by a distance-weighted (IDW-type) scheme for void filling and rasterization. Given the high ground-point density (~174 pts/m^2^, about 10 returns per cell), most grid cells were directly constrained by measured returns, so the resulting DEM is largely insensitive to the specific interpolation scheme. The 0.25 m cell size was chosen to match the micro-topographic scale of the regressive tidal creek and the UAV-LiDAR point density, providing sufficient resolution to capture its subtle erosion–deposition patterns. It should be noted, however, that in the intermittently inundated creek-bed cells, the lowest LiDAR returns correspond to the water surface rather than the bare bed; consequently, the DEM minimum elevation is tide-dependent (Section 4.4, Table 9), and these water-affected cells were excluded from the subsequent volumetric analysis.

**Figure 4:**
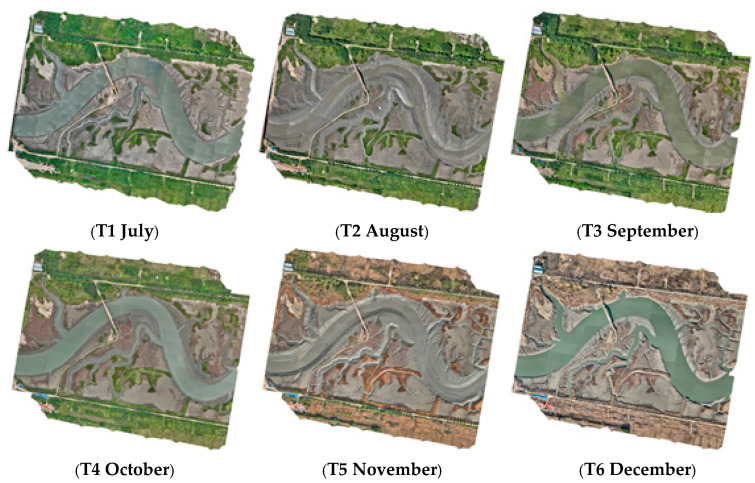
Monthly orthomosaic images acquired from July to December 2025 showing the temporal evolution of the regressive tidal channel and surrounding salt marsh at Sorae Wetland.

Figure 4 presents a time-series arrangement of orthomosaic images acquired monthly from July to December 2025, qualitatively illustrating the surface changes in the regressive tidal creeks and surrounding salt marshes in the Sorae Wetland. During the summer, surface moisture and vegetation cover around the tidal creeks are relatively prominent. In contrast, as autumn and winter progress, a decrease in vegetation density leads to a more distinct observation of the tidal creek boundaries. These results are considered to be closely related to the topographic and climatic characteristics of the study area. Topographically, approximately 84% of the Sorae Wetland lies at an elevation of about 2–5 m, with over 80% consisting of flat tidal flats with slopes of less than 2°. Originally, the Sorae Wetland was submerged by seawater during high tide; however, following reclamation projects around Sorae Port, the seawater inflow channels narrowed, transforming the area into a regressive salt marsh with a gradually decreasing flooding frequency. Furthermore, the wetland exhibits distinct moisture retention conditions and vegetation distributions driven by subtle elevation differences of several tens of centimeters. Since tidal creeks are situated at relatively lower elevations than the adjacent salt marshes, they create environments where moisture tends to accumulate.

In addition, the characteristics of the Korean monsoon climate reflect, to some extent, the geomorphological changes in the tidal creeks and surrounding salt marshes. Concentrated precipitation during the summer (June–August) induces an increase in surface moisture and salinity dilution, which promotes vegetation growth and increases cover. Consequently, the boundary contrast between the tidal creeks and the surrounding salt marshes tends to be relatively attenuated. Conversely, during autumn and winter, lower precipitation and falling temperatures result in vegetation senescence, reduced cover, and surface desiccation. This enhances the spectral and morphological contrast between vegetated and non-vegetated areas, making the boundaries of the tidal creeks appear more distinct than in the summer months.

While these orthomosaic images are useful for intuitively identifying planar positional changes and seasonal surface conditions of the tidal creeks, they have limitations in quantitatively analyzing actual ground elevations beneath vegetation or subtle erosion and deposition changes. Therefore, based on the visual change patterns identified in Figure 4, this study quantitatively characterized the micro-topographic changes in the tidal creeks through subsequent UAV LiDAR-based Digital Elevation Model (DEM) and DEM of Difference (DoD) analyses.

**Figure 5:**
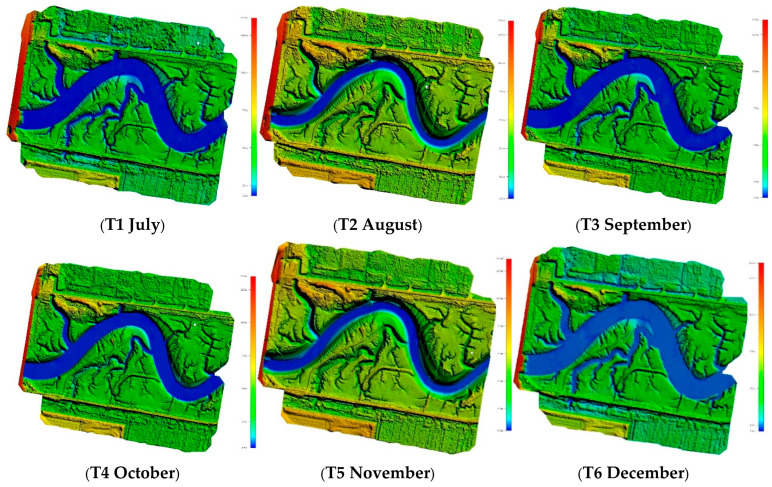
Monthly digital elevation models (DEMs) generated from UAV LiDAR data acquired from July to December 2025 (grid spacing 0.25 m).

Figure 5 presents the high-resolution DEMs generated from UAV LiDAR data acquired monthly from July to December 2025 in time-series format. All DEMs were produced applying the same processing workflow and grid spacing (0.25 m), thereby minimizing variance attributable to differences in resolution and processing conditions when comparing terrain between periods.

The generated DEM clearly illustrates the channel morphology, steep slopes, and vertical steps of the meandering tidal creeks, as well as the subtle elevation variations in the surrounding salt marshes. This effectively provides a quantitative reproduction of topographic features that are difficult to distinguish using ortho mosaic images alone. Particularly in vegetated sections, ground elevations were consistently extracted by leveraging the multi-return characteristics of LiDAR, which reliably reflect the actual geomorphic structure of the regressive tidal creeks. This time-series DEM dataset served as the essential foundational data for quantifying the spatial distribution and magnitude of erosion and deposition through subsequent DEM of Difference (DoD) analysis.

Table 3 summarizes the basic statistical characteristics of the UAV-LiDAR-derived DEMs (T1–T6). The overall mean elevation and standard deviation indicate that the broad topographic structure of the study area was consistently reproduced across the six surveys. However, the minimum elevation values varied substantially among survey dates. As demonstrated in Section 4.4, these DEM minimum values are strongly affected by the tide level at the time of flight in the intermittently inundated creek-bed zone. Therefore, the minimum elevation and raw low-elevation DoD values should not be interpreted directly as bare-earth erosion or deposition without considering tidal conditions.

### 4.2. Spatiotemporal Pattern Analysis of DEM Differences

#### 4.2.1. DEM of Difference

**Figure 6:**
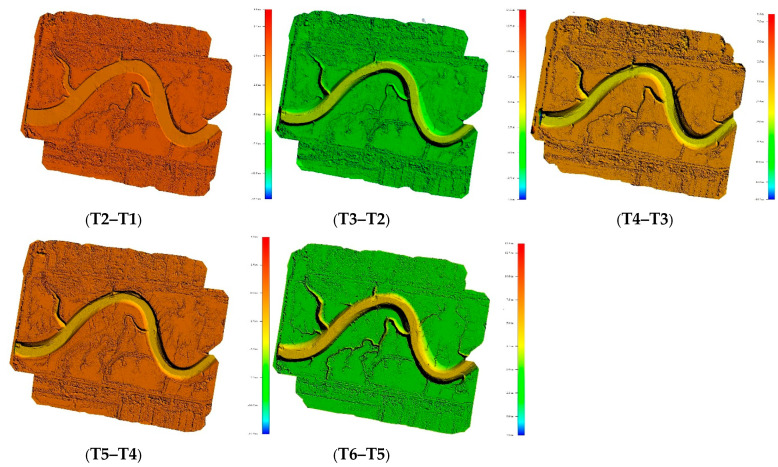
Monthly DEM of Difference (DoD) maps derived from successive UAV LiDAR-based DEMs (T2–T1, T3–T2, T4–T3, T5–T4, and T6–T5).

Figure 6 presents the DEM of Difference (DoD) results generated by differencing successive UAV LiDAR-based DEMs for adjacent time periods. To ensure analytical reliability, elevation changes within ±25 cm were excluded from interpretation. This threshold was adopted as a conservative minimum level of detection (minLoD) rather than as a simple match to the DEM grid spacing. Because all six surveys were acquired with the same platform, sensor, RTK processing and vertical datum, the dominant DoD uncertainty is the relative inter-epoch co-registration error rather than the absolute geolocation error. Following the propagated-error framework for repeat topographic surveys [43], in which the detection threshold is defined as minLoD = t·√(σ_1_^2^ + σ_2_^2^) at a given confidence level, the relevant per-DEM uncertainty (σ) here is the co-registration error of the RTK-based system. Given the sub-centimeter vertical precision of this system demonstrated in our previous work [13] and the consistency of the DEM statistics across the six epochs (Table 3), the propagated vertical uncertainty of the DoD is well below the adopted ±25 cm threshold. The ±25 cm value was therefore retained as a conservative minimum level of detection that also coincides with the 0.25 m DEM grid spacing, ensuring that only changes substantially exceeding the data uncertainty are interpreted. The DoD was generated using the QGIS Raster Calculator tool.

Examination of the DoD results reveals that erosion and deposition are not uniformly distributed across the study area but exhibit spatial patterns closely associated with tidal creek morphology. The tendency for erosion to concentrate on the outer slopes of the sinuous tidal creek can be explained by the outward migration of maximum velocity cores in typical meandering channels and the accompanying secondary (helical) flow [44,45]. This mechanism operates similarly in tidal creek systems dominated by tidal flows, but is modulated by tidal asymmetry [46].

The raw time-series DoD results appear to show that erosion and deposition are not continuously cumulative but occur intensively during specific periods before attenuating or reversing. As demonstrated in Section 4.4, however, much of this apparent temporal alternation in the low-elevation creek bed arises from the tide-dependent water surface rather than from genuine geomorphic change, so the raw DoD time series cannot by itself establish a nonlinear, threshold-controlled response. Any genuine morphological response that does occur could, in principle, involve feedback such as hysteresis, in which antecedent erosion and deposition processes govern subsequent responses, and hydro-morphodynamic adjustment of flow velocity and shear stress following morphological change—but the present water-affected DoD time series is not sufficient to confirm these mechanisms, which remain hypotheses for future water-corrected or low-tide-synchronized monitoring.

#### 4.2.2. Statistical Analysis by 1 m Elevation Section

To identify the elevation intervals where erosion and deposition predominantly occur, this study analyzed the time-series DEM data (T1–T6) by dividing them into 1 m elevation bands (Table 4, Table 5 and Table 6). For this purpose, the DEMs were reclassified by elevation, resulting in the generation of raster maps that indicate the specific elevation interval to which each grid cell belongs.

In consideration of the topographic characteristics and the spatial distribution of the tidal creeks, these elevation zones were defined at 1 m intervals. This categorization provides a spatial framework for quantitatively analyzing whether erosion and deposition are concentrated within specific elevation ranges. The intervals were established to comprehensively encompass the terrain from the lower tidal creek beds to the higher salt marsh areas, facilitating a systematic comparison of how geomorphic changes vary by elevation over time.

To quantify the characteristics of change for each elevation interval, zonal statistics were performed on the DEM of Difference (DoD) using the 1 m reclassified elevation rasters as a baseline. Through this process, the area affected by erosion and deposition, total volume changes, and mean depth of change were calculated for each elevation interval. However, to eliminate the influence of artificial structures such as the bridge located on the left side of the study area, intervals exceeding an elevation of 10 m were excluded from the analysis.

**Figure 7:**
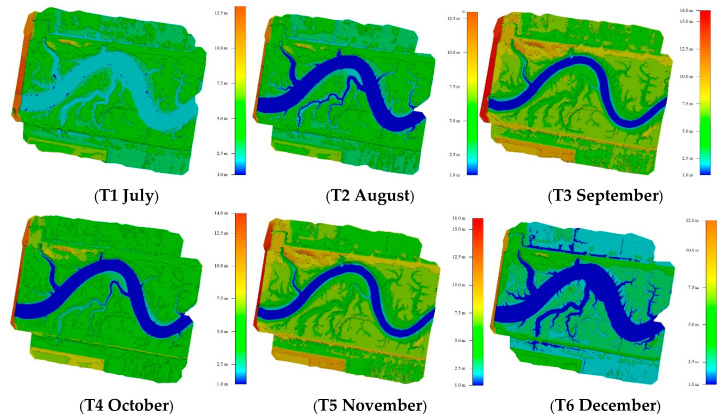
Reclassified DEM maps divided into 1 m elevation intervals. Used as the spatial framework for analyzing elevation-dependent erosion and deposition patterns.

Figure 7 presents the monthly results of the reclassified DEMs into 1 m elevation intervals to quantitatively analyze erosion and deposition characteristics relative to elevation. Each color represents a distinct elevation band, which served as the spatial framework for the subsequent zonal statistics and volumetric change analysis. This reclassification enabled a systematic comparison to determine whether erosion and deposition were concentrated within specific elevation ranges. In particular, by distinguishing between the low-elevation intervals corresponding to the tidal creek beds and the higher-elevation salt marsh zones, this approach provided a basis for analyzing how the micro-topographic changes in the regressive tidal creeks manifest differentially according to elevation.

To quantify the geomorphic changes, the volumetric change was calculated by deriving the arithmetic mean of the DoD values for each elevation interval and multiplying it by the total area of the corresponding zone. The equations for the two most critical indicators—mean change depth and total volume—are given by Equations (1)–(3). First, the mean change depth represents the average increase or decrease in elevation across all pixels within a specific elevation interval.

(1) $$∆h_{avg}=\frac{1}{n}\sum_{i=1}^{n}(Z_{T2,i}-Z_{T1,i})$$

- n: number of pixels in the elevation interval
- $Z_{Tk+1,\;i}$, $Z_{Tk,i}$: elevation of the cell i (later/earlier survey)

The total erosion and deposition volumes are determined by calculating the actual three-dimensional volume through the product of unit area and elevation change, which is equivalent to the (mean change depth × total area of the interval).

(2) $$V_{total}=\sum_{i=1}^{n}({∆h}_{i}\times A_{pixel})$$

(3) $$V_{total}=∆h_{avg}\times n\times A_{pixel}=∆h_{avg}\times A_{zone}$$

- $\Delta h_{i}\;=\;Z_{Tk+1,i}\;-\;Z_{Tk,i}$: DoD value of cell
- $\Delta h_{avg}$: mean change depth of the interval
- $A_{pixel}$: ($0.25\;m\times 0.25\;m\;=\;0.0625{\;m}^{2}$)
- $A_{zone}\;=\;n\times A_{pixel}$: total area of the interval

Table 4, Table 5 and Table 6 summarize the area, mean erosion and deposition depth, and total volume for each 1 m elevation interval by period, based on the DEM of Difference (DoD) analysis. These tables facilitate a quantitative comparison of the elevation ranges where erosion and deposition occur, as well as their characteristic temporal variations.

The zonal statistics show that the largest apparent elevation changes are concentrated in the lowest elevation intervals. During the T2–T1 period, the lowest range (−2.0 m to 2.0 m) registered an apparent mean decrease of up to 3.77 m, and during the T3–T2 period the 1.0–2.0 m range registered an apparent mean increase of up to 2.08 m. As detailed in Section 4.4, however, the lowest creek-bed cells are intermittently inundated, and the LiDAR returns there correspond to the water surface rather than to the bare creek bed. Because the DEM minimum of each survey tracks the tide level at the time of flight, these apparent low-zone changes are dominated by inter-survey differences in water-surface elevation and cannot be interpreted as genuine bed erosion or deposition. They are therefore reported here as raw zonal values but are excluded from the quantitative sediment budget. By contrast, the higher, consistently subaerial intervals exhibit only small mean changes (generally within a few centimeters per interval); these subaerial changes—rather than the tide-contaminated low-zone values—provide the reliable basis for the volumetric analysis presented in Section 4.4.

In summary, the time-series geomorphic change patterns in the study area can be interpreted as the combined result of differences in tidal inundation frequency relative to elevation and the resulting spatial heterogeneity in flow velocity and shear stress distribution. Specifically, low-elevation areas exhibit significant geomorphic changes due to repetitive inundation and velocity fluctuations, whereas high-elevation areas show relatively minor changes due to lower inundation frequency. This supports the premise that regressive tidal creeks are dynamic geomorphic systems that are continuously readjusting within the balance of energy conditions and sediment supply.

The period-to-period volumetric results were further summarized to evaluate the overall geomorphic balance during the six-month monitoring period(see Table 7). In this raw budget, which still includes the water-affected creek-bed zone, the largest apparent period-to-period swings occurred during T2–T1 (−130,252 m^3^) and T6–T5 (+142,296 m^3^); as shown in Section 4.4, however, these swings are dominated by the difference in tide level between the paired surveys rather than by genuine sediment turnover. After the water-affected zone is removed, the corrected subaerial budget presented in Section 4.4 yields a cumulative net change of −26,525 m^3^ over the six-month period—a modest net lowering—rather than the apparent +100,149 m^3^ net deposition implied by the raw budget. The corrected figures should therefore be used for geomorphic interpretation.

### 4.3. Cross-Sectional Profile Analysis

**Figure 8:**
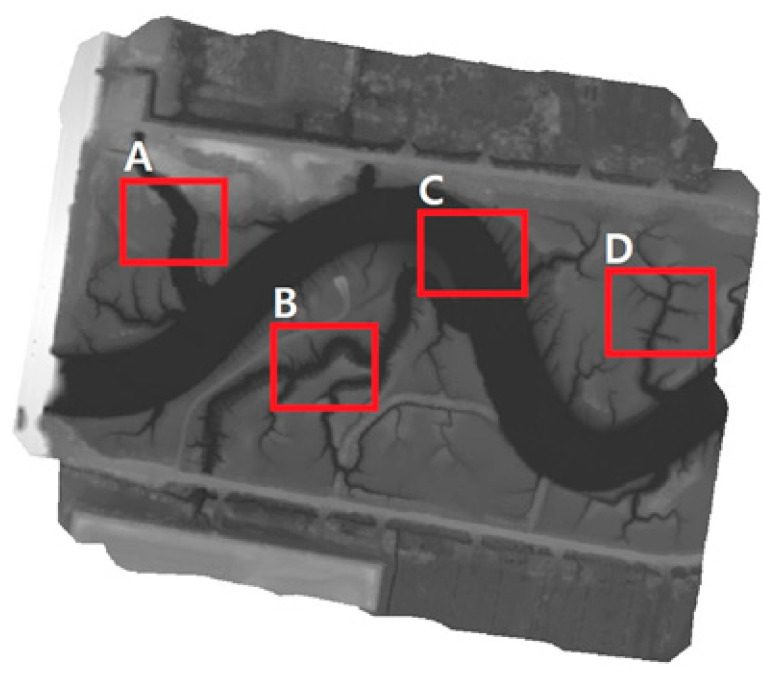
Locations of cross-sectional profile analysis points (A–D) within the regressive tidal channel.

**Figure 9:**
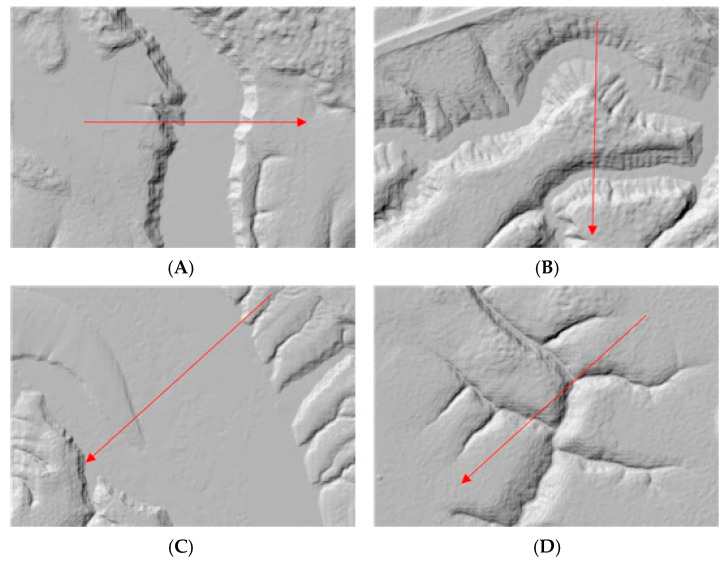
Cross-sectional transect lines used for elevation profile analysis (**A**–**D**).

To verify how micro-topographic changes in the tidal creek manifest in actual terrain cross-sections rather than as spatially averaged outcomes, cross-sectional profile analyses were conducted across the tidal creek. Four locations (A, B, C, D) within the study area showing distinct topographic changes were selected (Figure 8), and identical cross-sectional transects were established at each location (Figure 9) to compare elevation changes over the six-month period in time-series format. This transect-based analysis serves to validate the preceding DoD analysis and elevation-zone erosion–deposition results at a site-specific level.

**Figure 10:**
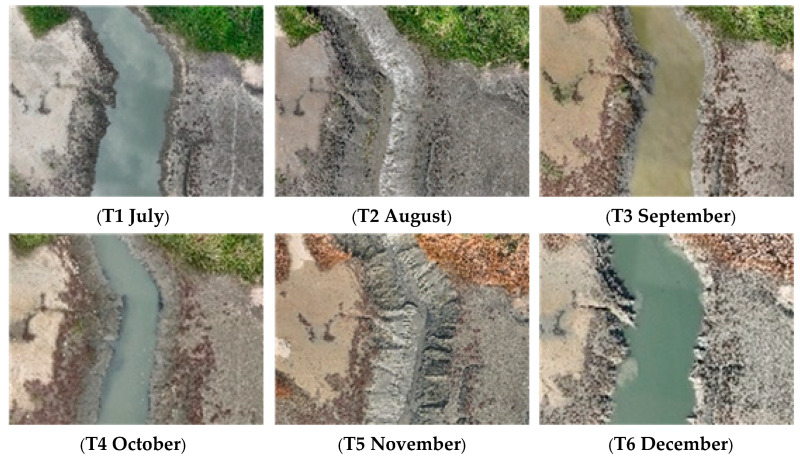
Micro-topographic changes in critical zone A.

**Figure 11:**
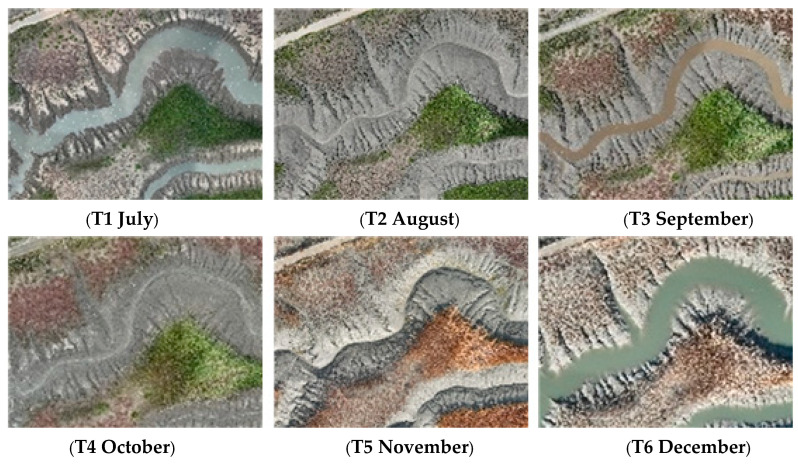
Micro-topographic changes in critical zone B.

**Figure 12:**
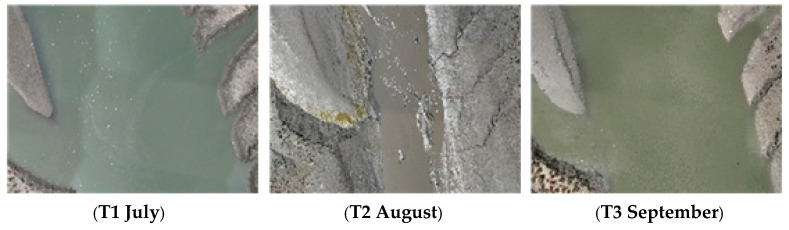

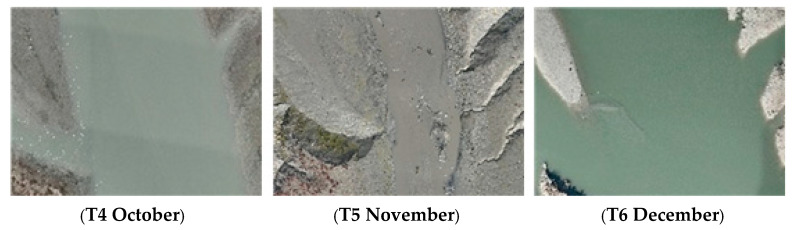
Micro-topographic changes in critical zone C.

**Figure 13:**
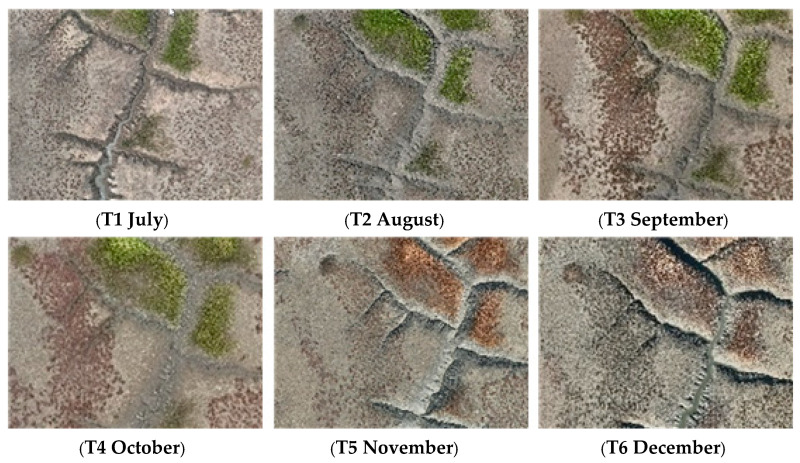
Micro-topographic changes in critical zone D.

Figure 10, Figure 11, Figure 12 and Figure 13 present time-series orthomosaic images extracted from UAV LiDAR data repeatedly observed over a six-month period for the four selected study sites (A–D). Each figure overlays the monthly changes at the same cross-sectional location, allowing for a direct visualization of the actual patterns of micro-topographic changes.

**Figure 14:**
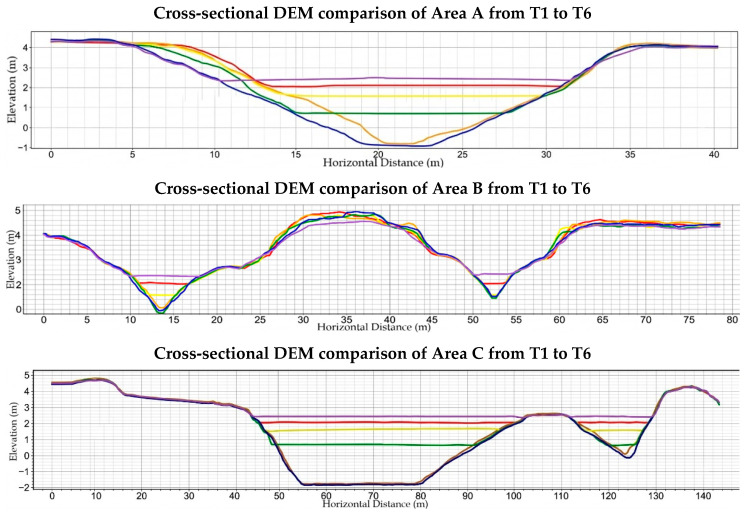

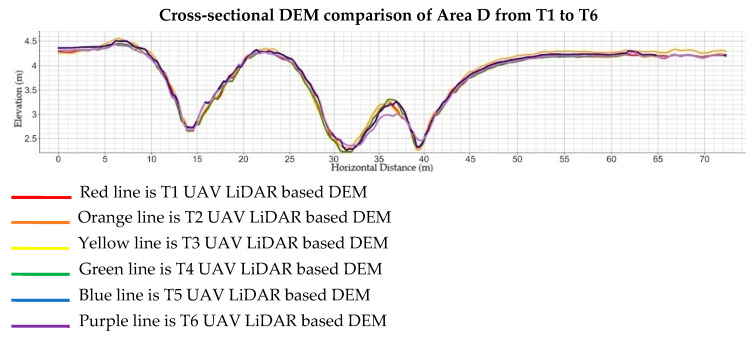
Cross-sectional elevation profiles derived from UAV-LiDAR DEMs for periods T1–T6 along transects A–D. The x-axis represents distance along each cross-section (m), and the y-axis represents elevation (m). (The cross-sectional transect lines are shown in Figure 9 as the red arrows.)

Figure 14 compares the elevation profiles extracted for the four cross-sections (A–D) across the tidal creeks, comprehensively illustrating both the differences in micro-topographic changes between sections and their common characteristics. Since each profile was derived using the same analysis period and methodology, it is possible to directly compare the spatial heterogeneity of the geomorphic changes occurring within the tidal creeks.

At Site A, distinct cycles of erosion and deposition were observed, primarily centered on the creek bed and the outer slopes. In particular, an erosional pattern characterized by a rapid decrease in elevation at the bottom of the creek appeared during the summer, followed by partial deposition in the same section, indicating a tendency for the cross-sectional shape to readjust. These changes are consistent with the results of the DoD and elevation-based analyses, which showed that erosion and deposition are concentrated in low-elevation intervals.

At Site B, periodic elevation changes were also identified around the creek bed, though the magnitude of change was relatively moderate compared to Site A. While repetitive erosion was observed on the outer slopes, deposition predominated on the inner slopes, maintaining the asymmetry of the cross-section. This suggests that flow velocity and shear stress act spatially unevenly within the meandering tidal creeks.

In the case of Site C, the overall cross-sectional morphology remained relatively stable; however, subtle elevation changes were found to accumulate or mitigate over time along the creek slopes. Specifically, while erosion occurred repeatedly on the outer slopes, intermittent deposition appeared in certain sections of the creek bed, indicating that local geomorphic adjustments are taking place.

At Site D, the cross-sectional profile exhibited the highest degree of morphological stability among the four study sites. Elevation changes over the six-month observation period were generally limited in magnitude, with no pronounced erosional scour or depositional infilling observed at the creek bed. Minor but consistent adjustments were identified along the upper slope margins, where slight lateral erosion occurred during the summer period (T1–T2) and partially recovered through low-magnitude deposition in the autumn months (T4–T5). The relatively subdued geomorphic response at Site D is attributed to its position within a low-curvature reach of the tidal creek, where tidal flow velocities and associated shear stresses are comparatively reduced. This pattern is broadly consistent with that observed at Site C, suggesting that reaches with lower channel curvature and gentler slope gradients experience attenuated geomorphic dynamics relative to the more actively adjusting sections represented by Sites A and B.

The comparison results show that in all cross-sections, geomorphic changes tended to concentrate at the center of the creeks and along the slope boundaries; however, the scale and pattern of these changes varied distinctly depending on the cross-sectional location. In sections A and B, repeated erosion and deposition around the creek bed resulted in relatively large fluctuations, whereas in sections C and D, the overall cross-sectional forms remained comparatively stable, with primarily localized elevation adjustments observed.

These cross-sectional variations are interpreted to be attributed to differences in the curvature of the tidal creeks, slope morphology, and local hydrological conditions. This implies that geomorphic changes within regressive tidal creeks do not occur uniformly throughout the study area but are instead locally concentrated along the creek structures. Figure 14 clearly demonstrates that the micro-topographic changes in regressive tidal creeks manifest as spatially uneven and non-linear responses.

### 4.4. Comparison of Tidal Conditions and Micro-Topographic Changes

One-minute-interval tide level data from the Incheon Songdo tide gauge station constitute a high-resolution dataset well-suited for analyzing micro-topographic changes on tidal flats near Sorae Wetland. As Sorae Wetland has an extremely large tidal range and well-developed complex tidal creek networks, these data were utilized to examine the causes of topographic change.

**Figure 15:**
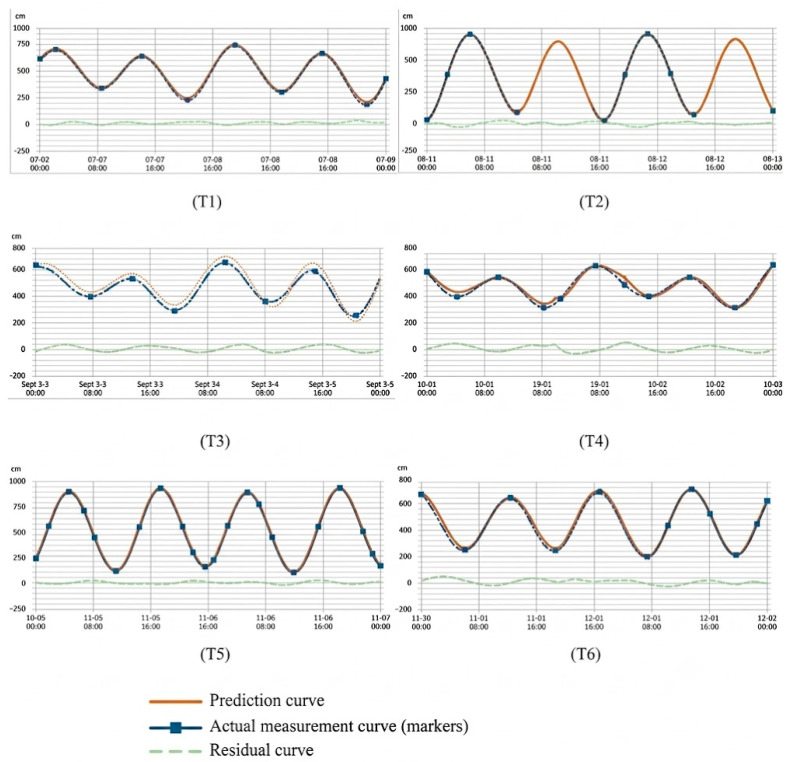
Predicted tide level, observed tide level, and residual component recorded at 1 min intervals at the Incheon Songdo tide gauge station during the 24 h period before and after each UAV-LiDAR survey. The x-axis represents time, and the y-axis represents tide level in centimeters.

Figure 15 illustrates the tide level curves at 1 min intervals recorded at the Incheon Songdo tidal station for the 24 h periods surrounding each UAV LiDAR survey date. The figure compares monthly tidal levels during the study period through predicted and observed values, while isolating the difference between the two as a residual component. To ensure the curves remain distinguishable in both color and greyscale, the predicted tide level (solid line) was calculated via tidal harmonic analysis, the actual observed tide level measured in the field is shown as a dashed line, and the residual is shown as a dotted line. The residual represents the water level fluctuations not accounted for by tidal predictions, reflecting the influence of short-term meteorological conditions or local oceanic factors.

Overall, the predicted and observed tide levels showed similar temporal patterns throughout the study period, indicating qualitative agreement and confirming that tidal variations were stably reproduced. This indicates that the tidal prediction data used in this study possesses sufficient reliability as a reference for analyzing micro-topographic changes in tidal creeks. Meanwhile, temporary increases in the residual component are observed at specific times, suggesting that water levels may have been momentarily amplified by short-term external forcing, such as strong winds or the passage of low-pressure systems.

The residual component shown in Figure 15 was used primarily to identify short-term deviations between predicted and observed tide levels during each UAV-LiDAR survey. These deviations provide contextual information for interpreting whether low-elevation DEM differences may have been influenced by transient water-level anomalies. Therefore, the tide-level records were used not as direct evidence of erosion or deposition, but as auxiliary information for distinguishing tide-dependent apparent elevation change from more reliable subaerial geomorphic change.

Table 8 records the tide levels at 10 min after the completion of each UAV survey, accounting for a 10 min tidal delay for seawater to travel from the Incheon Songdo tidal station to the study area near Sorae Wetland.

To test whether the large apparent elevation changes in the lowest creek-bed zone represent genuine geomorphic change, the DEM minimum elevation (Min) of each survey was compared with the tide level recorded at the time of the corresponding flight (Table 9). The DEM minimum decreases monotonically with falling tide level: it is highest at the high-tide surveys (T6, +2.09 m at 688 cm; T1, +1.82 m at 656 cm) and lowest at the low-tide surveys (T2, −1.92 m at 219 cm; T5, −1.97 m at 9 cm). The change in the DEM minimum between T1 and T2 (+1.82 m to −1.92 m, a difference of about −3.7 m) corresponds almost exactly to the apparent −3.77 m decrease reported for that period in Section 4.2.2. This confirms that the lowest DEM returns in the intermittently inundated creek bed represent the instantaneous water surface rather than the bare bed, and that DoD differences in this zone are controlled by inter-survey tide differences rather than by sediment erosion or deposition. Accordingly, the water-affected creek-bed zone was excluded from the volumetric budget, and only the consistently subaerial surface was retained for the quantitative analysis (Table 10).

## 5. Discussion

This study employed repeated UAV-LiDAR observations to analyze micro-topographic change in a vegetation-covered regressive tidal creek. The results show that raw DoD patterns in regressive tidal creeks can appear highly nonlinear when the lowest creek-bed zones are intermittently inundated. However, after accounting for tide-dependent water-surface returns, the consistently subaerial surface showed more modest geomorphic change over the six-month monitoring period. This finding indicates that large apparent elevation differences in the lowest creek-bed zone should not be interpreted directly as rapid bed erosion or deposition without considering tide level at the time of UAV-LiDAR acquisition. Instead, the integration of UAV-LiDAR-derived DEMs with tide-level information is essential for distinguishing tide-dependent apparent elevation change from more reliable subaerial geomorphic change in tidal wetland environments.

Regressive tidal flats are generally understood to undergo gradual elevation rise during terrestrialization [37]. The DoD analysis initially appeared to contradict this, showing apparent vertical changes exceeding 2 m in the lower reaches of the creek between July and September (T1–T2–T3). As shown in Section 4.4, however, this apparent rapid alternation coincides with the high–low–high sequence of tide levels at the T1, T2 and T3 surveys and is an artifact of the tide-dependent water surface rather than evidence of nonlinear geomorphic evolution. After this effect is removed, the subaerial record is consistent with the gradual adjustment expected for a terrestrializing regressive tidal flat.

In particular, the maximum apparent decrease of 3.768 m in the lowest creek-bed zone during the T2–T1 period was initially interpreted as rapid erosion. However, this apparent change coincides with the large fall in tide level between the two surveys (656 cm at T1 versus 219 cm at T2), and—as shown in Section 4.4 (Table 9)—the DEM minimum tracks the tide-dependent water surface in the creek bed. The apparent 3.768 m change therefore reflects the difference in water-surface elevation between the two flights rather than genuine bed-level erosion, and it is excluded from the sediment budget. This result highlights a methodological caveat for UAV-LiDAR monitoring of regressive tidal creeks: in intermittently inundated channels the lowest DEM returns represent the water surface, so apparent elevation changes in this zone must be separated from genuine bed-level change before geomorphic interpretation. Once the water-affected zone is excluded, the consistently subaerial surface shows only modest net change over the six-month period (Table 10), and the bare-earth cross-sections in Section 4.3 likewise indicate gradual, low-magnitude adjustment rather than abrupt large-scale scour. The genuine geomorphic signal of the regressive tidal creek is therefore comparatively subtle, consistent with its limited hydrological connectivity and infrequent inundation.

Similarly, the apparent 2.075 m of deposition in the lowest zone during the T3–T2 period corresponds to the rise in tide level between these surveys (219 cm at T2 versus 589 cm at T3) and is likewise attributable to the changing water surface rather than to sediment infilling. These paired examples illustrate that, without separating the water-surface signal, repeated UAV-LiDAR surveys of intermittently inundated creeks can produce spurious erosion–deposition cycles of several meters. Where genuine morphological adjustment does occur, it is consistent with prior research [47], which posits that tidal channels and adjacent wetlands are reconfigured through the interaction of hydrodynamic conditions and sediment supply.

Once the tide-dependent water-surface signal is removed, UAV-LiDAR monitoring shows that the regressive tidal flat undergoes comparatively modest and gradual short-term change, consistent with its limited inundation and ongoing terrestrialization. At the same time, the results indicate that a more frequent occurrence of extreme precipitation events under climate change scenarios could still increase the geomorphic instability of regressive tidal creeks [48], further underscoring the importance of high-precision monitoring.

## 6. Conclusions

This study used six repeated UAV-LiDAR surveys to quantify seasonal micro-topographic changes in a regressive tidal creek at Sorae Wetland and to evaluate how erosion and deposition varied across elevation zones, channel sections, and tidal conditions. The main conclusions are as follows.

First, UAV-LiDAR effectively overcame the limitations of conventional optical surveying and digital topographic maps in a vegetation-dense regressive tidal-flat environment. The Penta Return mode provided high-density point cloud data averaging 174 pts/m^2^ and enabled the generation of 0.25 m resolution DEMs suitable for detecting micro-topographic changes in the tidal creek.

Second, the DoD analysis showed that the largest apparent elevation changes occurred in the lowest creek-bed zone, but these were found to be controlled by the tide-dependent water-surface return rather than by genuine bed erosion, as the DEM minimum of each survey scaled monotonically with the tide level at the time of flight (Table 9). After excluding this water-affected zone, the consistently subaerial surface showed only modest net change over the six-month period. This result demonstrates the importance, in regressive tidal creeks, of distinguishing tide-dependent water-surface signals from genuine bed-level change when interpreting repeated UAV-LiDAR surveys.

Third, the comparison with tide-level data showed that the apparent low-zone elevation changes are governed mainly by the tide level at the time of each flight, while residual water-level variations and meteorological forcing (including rainfall-related runoff) may additionally affect water levels. Both tidal and meteorological factors should therefore be considered when interpreting short-term UAV-LiDAR surveys of regressive coastal wetlands.

From a management perspective, the repeated UAV-LiDAR and DoD approach used in this study can support the early detection of erosion-prone creek beds and unstable bank sections in regressive tidal flats. Such information is useful for prioritizing field inspections, planning wetland restoration, and assessing geomorphic damage after extreme rainfall or storm events. The results also show that high-resolution time-series monitoring can help distinguish short-term localized disturbance from the long-term terrestrialization trend of coastal wetlands.

Future research should extend the monitoring period to one year or longer to account for vegetation life cycles and seasonal hydrological variability. Additional field measurements of flow velocity, sediment properties, and vegetation density would also improve the interpretation of the critical conditions that trigger nonlinear topographic changes in regressive tidal creeks.

## Figures and Tables

**Table 1:** Flight plan and LiDAR payload settings.

UAV Flight Plan		LiDAR Payload Setting	
Flight Height	100 m	Reflect Mode	Penta
Side Overlap (RGB)	60%	Sampling Rate	240 kHz
Front Overlap (RGB)	70%	Scan Mode	Repeated
Area Covered	0.28 km^2^	Picture Mode	Interval
Average GSD	2.93 cm/pix	Point Cloud Density	174 pts/m^2^

**Table 2:** Observation dates and weather conditions for each LiDAR survey.

No	Date	Avg Temp	High Temp	Low Temp	Weather
T1	8 July 2025	30.0 °C	35.6 °C	25.2 °C	Few clouds
T2	12 August 2025	27.7 °C	32.6 °C	22.4 °C	Few clouds
T3	4 September 2025	25.7 °C	28.7 °C	23.0 °C	Few clouds
T4	2 October 2025	21.1 °C	24.2 °C	17.9 °C	Cloudy
T5	6 November 2025	12.6 °C	17.1 °C	8.6 °C	Few clouds
T6	1 December 2025	4.9 °C	11.2 °C	2.0 °C	Clear

**Table 3:** Summary statistics of UAV LiDAR-derived DEM raster layers for each observation period.

No	Min	Max	Range	Sum	Mean	Std_Dev	Sum of Squares
T1	1.8166	13.6515	11.8348	24,584,974	4.1907	1.8924	21,010,405
T2	−1.9228	13.7481	15.6710	22,526,072	3.8118	2.5145	37,365,680
T3	1.4412	13.5156	12.0744	22,101,026	3.9757	1.6865	15,812,898
T4	0.5362	13.5108	12.9746	21,292,764	3.8401	1.8894	19,795,865
T5	−1.9742	13.5832	15.5575	20,224,405	3.6114	2.3376	30,601,984
T6	2.0914	13.5205	11.4291	22,471,371	4.0508	1.5441	13,227,639

**Table 4:** Elevation-based reclassification results: T2–T1 (unit: m).

Zone				T2–T1		
Min	Max	Class	Zone	Area (m^2^)	Mean	Volume (m^3^)
−2.0	−1.0	1	−2.0~−1.0	27,938.13	−3.77	−105,281.81
−1.0	0.0	2	−1.0~0.0	8280.63	−2.65	−21,959.30
0.0	1.0	3	0.0~1.0	8351.50	−1.59	−13,293.40
1.0	2.0	4	1.0~2.0	11,580.63	−0.55	−6312.62
2.0	3.0	5	2.0~3.0	22,916.38	+0.04	+998.49
3.0	4.0	6	3.0~4.0	91,823.25	+0.02	+1741.54
4.0	5.0	7	4.0~5.0	125,496.19	0.07	+9142.02
5.0	6.0	8	5.0~6.0	28,379.38	+0.07	+2063.54
6.0	7.0	9	6.0~7.0	19,501.75	+0.10	+1994.20
7.0	8.0	10	7.0~8.0	9031.50	+0.09	+783.52
8.0	9.0	11	8.0~9.0	1041.25	−0.03	−34.42
9.0	10.0	12	9.0~10.0	625.75	−0.15	−94.19

**Table 5:** Elevation-based reclassification results: T3–T2 and T4–T3 (unit: m).

T3–T2				T4–T3		
Zone	Area (m^2^)	Mean	Volume (m^3^)	Area (m^2^)	Mean	Volume (m^3^)
1.0~2.0	55,246.13	+2.08	+114,678.57	11,415.31	−0.18	−2078.12
2.0~3.0	23,392.56	−0.08	−1978.13	23,325.69	−0.01	−348.71
3.0~4.0	97,640.38	−0.08	−7430.55	98,081.00	−0.02	−1737.04
4.0~5.0	111,124.88	−0.06	−7133.61	109,022.13	−0.01	−1130.74
5.0~6.0	26,974.75	−0.06	−1529.71	26,790.56	−0.02	−601.56
6.0~7.0	17,908.75	−0.07	−1294.19	17,396.94	0.00	−23.05
7.0~8.0	8318.50	−0.03	−291.06	8560.06	0.00	+15.84
8.0~9.0	928.25	−0.20	−184.26	919.19	0.00	−4.22
9.0~10.0	507.13	−0.38	−194.40	534.50	−0.06	−33.34

**Table 6:** Elevation-based reclassification results: T5–T4 and T6–T5 (unit: m).

T5–T4				T6–T5		
Zone	Area (m^2^)	Mean	Volume (m^3^)	Area (m^2^)	Mean	Volume (m^3^)
2.0~3.0	24,008.19	0.00	+103.88	80,855.69	+1.99	+160,921.38
3.0~4.0	97,060.88	−0.03	−2689.44	109,032.75	−0.07	−7343.82
4.0~5.0	110,280.94	+0.02	+1975.59	98,950.25	−0.06	−6415.51
5.0~6.0	26,154.06	−0.01	−131.71	26,214.75	−0.10	−2491.91
6.0~7.0	17,718.94	+0.03	+479.45	15,931.63	−0.10	−1623.68
7.0~8.0	8902.50	−0.01	−77.22	7219.19	−0.09	−614.70
8.0~9.0	917.94	−0.15	−141.81	843.25	−0.11	−94.05
9.0~10.0	596.81	−0.19	−114.28	409.38	−0.10	−42.13

**Table 7:** Period-wise and cumulative volumetric change in the regressive tidal creek derived from the raw DoD analysis, including the intermittently inundated creek-bed zone (July–December 2025). These values are affected by the tide-dependent water surface (Section 4.4) and are superseded, for geomorphic interpretation, by the water-corrected subaerial budget in Section 4.4.

Period	Month	Erosion (m^3^)	Deposition (m^3^)	Net Change (m^3^)	Cumulative Net (m^3^)
T2–T1	July–August	−146,976	+16,723	−130,252	−130,252
T3–T2	August–September	−20,036	+114,679	+94,643	−35,609
T4–T3	September–October	−5957	+16	−5941	−41,550
T5–T4	October–November	−3154	+2559	−596	−42,146
T6–T5	November–December	−18,626	+160,921	+142,296	+100,149
**Total**	**Jul** **y–** **Dec** **ember**	**−194,749**	**+294,898**	**+100,149**	**—**

Note: Positive values denote deposition and negative values denote erosion. These figures are derived from the raw DoD and include the intermittently inundated creek-bed zone, whose apparent changes are dominated by the tide-dependent water surface (Section 4.4); the large period-to-period swings therefore largely reflect differences in tide level between surveys rather than genuine sediment turnover. The water-corrected subaerial budget is reported separately in in Section 4.4 and supersedes these values for geomorphic interpretation.

**Table 8:** Tide level data for each observation period.

No	Date	Flight Time	Correction Time	Tide Level (cm)	Station
T1	8 July 2025	14:40~14:50	15:00	656	Incheon Songdo
T2	12 August 2025	14:10~14:20	14:30	219	Incheon Songdo
T3	4 September 2025	14:50~15:00	15:10	589	Incheon Songdo
T4	2 October 2025	13:20~13:30	13:40	526	Incheon Songdo
T5	6 November 2025	11:10~11:20	11:30	9	Incheon Songdo
T6	1 December 2025	13:40~13:50	14:00	688	Incheon Songdo

**Table 9:** Relationship between the observed tide level at the time of each UAV-LiDAR survey and the corresponding DEM minimum elevation (Min). Surveys are ordered by descending tide level; the DEM minimum decreases monotonically with falling tide level, indicating that the lowest DEM values represent the tide-dependent water surface in the creek bed rather than the bare-earth elevation.

Tide Level Sequence	T5 (9 cm)	T2 (219 cm)	T4 (526 cm)	T3 (589 cm)	T1 (656 cm)	T6 (688 cm)
DEM Min (m)	−1.97	−1.92	+0.54	+1.44	+1.82	+2.09

**Table 10:** **(revised).** Subaerial volumetric change in the regressive tidal creek after excluding the intermittently inundated (water-affected) creek-bed zone (July–December 2025).

Period	Month	Erosion (m^3^)	Deposition (m^3^)	Net (m^3^)	Cumulative (m^3^)
T2–T1	July~August	−129	+16,723	+16,595	+16,595
T3–T2	August~September	−20,036	0	−20,036	−3441
T4–T3	September~October	−3879	+16	−3863	−7304
T5–T4	October~November	−3154	+2559	−596	−7900
T6–T5	November~December	−18,626	0	−18,626	−26,525
Total		−45,823	+19,298	−26,525	

Note: Volumes are restricted to the consistently subaerial surface. The intermittently inundated creek-bed zone is excluded because its apparent elevation changes are dominated by the tide-dependent water-surface return (see Section 4.1) and cannot be unambiguously separated from genuine bed-level change. The excluded zone is exactly where real creek-bed scour and infilling are also expected to be most active; the subaerial budget therefore represents a conservative lower bound on total geomorphic turnover and should be interpreted alongside the qualitative cross-sectional evidence in Section 4.3.

## Data Availability

Data is available from the corresponding author upon reasonable request.
